# CARD8 is a negative regulator for NLRP3 inflammasome, but mutant NLRP3 in cryopyrin-associated periodic syndromes escapes the restriction

**DOI:** 10.1186/ar4483

**Published:** 2014-02-12

**Authors:** Sayaka Ito, Yukichi Hara, Tetsuo Kubota

**Affiliations:** 1 Division of Biomedical Laboratory Sciences, Tokyo Medical and Dental University Graduate School of Health Care Sciences, 1-5-45 Yushima, Bunkyo-ku, Tokyo 113-8510, Japan

## Abstract

**Introduction:**

NLRP3 plays a role in sensing various pathogen components or stresses in the innate immune system. Once activated, NLRP3 associates with apoptosis-associated speck-like protein containing a caspase recruitment domain (ASC) and procaspase-1 to form a large protein complex termed inflammasome. Although some investigators have proposed a model of NLRP3-inflammasome containing an adaptor protein caspase recruitment domain-containing protein 8 (CARD8), the role of this molecule remains obscure. This study aimed to clarify the interaction between CARD8 and wild-type NLRP3 as well as mutant forms of NLRP3 linked with cryopyrin-associated periodic syndromes (CAPS).

**Methods:**

In here HEK293 expression system, cells were transfected with the cDNAs for inflammasome components. Also used were peripheral blood mononuclear cells (PBMCs) and human monocyte-derived macrophages (HMDMs) from healthy volunteers. The interaction of CARD8 and NLRP3 was studied by immunoprecipitation. The effect of CARD8 expression on IL-1β secretion was assessed by ELISA. CARD8 knockdown experiments were carried out by transfection of the specific siRNA into HMDMs.

**Results:**

In HEK293 cells, CARD8 interacted with wild-type NLRP3, but not with CAPS-associated mutant NLRP3. CARD8 significantly reduced IL-1β secretion from cells transfected with wild-type NLRP3, but not if they were transfected with mutant NLRP3. In addition, association of endogenously expressed CARD8 with NLRP3 was confirmed in resting PBMCs, and CARD8 knockdown resulted in higher amount of IL-1β secretion from HMDMs.

**Conclusions:**

Until specific stimuli activate NLRP3, CARD8 holds NLRP3, and is supposed to prevent activation by subtle stimuli. However, CAPS-associated mutant NLRP3 is unable to bind with CARD8, which might be relevant to the pathogenesis of CAPS.

## Introduction

The NLR (nucleotide binding oligomerization domain-like receptor) protein family plays a role as an intracellular sensor for pathogens and cell injury by detecting conserved structures, such as pathogen-associated molecular patterns (PAMPs) and damage-associated molecular patterns (DAMPs) [1,2]. NLRP3, a member of the NLR, is a cytoplasmic protein expressed predominantly in monocytes and macrophages, and forms a caspase-1 activating multiprotein complex termed inflammasome with other proteins, including an adaptor protein ASC (apoptosis-associated speck-like protein containing a caspase recruitment domain (CARD)) and procaspase-1 [3,4]. The NLRP3 is composed of three domains referred to as pyrin domain (PYD) at the N-terminus, leucine-rich repeats (LRRs) at the C-terminus, and nucleotide binding-oligomerization domain (NOD) in the middle. When PAMPs or DAMPs are recognized by the LRRs, NLRP3 oligomerizes by self-association through the NOD, and binds with ASC via their PYD domains [5,6]. ASC and procaspase-1 bind with each other through their CARD [7,8], which leads to the assembly and autocleavage of the procaspase-1 to produce active caspase-1. Then caspase-1 processes proIL-1β to mature IL-1β, which is released into the extracellular space [9].

Mutations in the *cold*-*induced autoinflammatory syndrome 1* (*CIAS1*) gene encoding NLRP3 result in cryopyrin-associated periodic syndromes (CAPS) characterized by recurrent episodes of systemic inflammatory attacks in the absence of infection or autoimmune diseases [10]. CAPS include three clinical entities: familial cold autoinflammatory syndrome (FCAS), Muckle-Wells syndrome (MWS) and chronic infantile neurologic cutaneous articular syndrome (CINCA). The mildest form, FCAS, presents with a cold-induced urticarial rash, fever and arthralgia. CINCA, the most severe form of CAPS, includes neonatal-onset high fever, aseptic meningitis, sensory hearing loss, papilledema, arthritis with bone overgrowth and secondary amyloidosis. MWS is the intermediate phenotype, and overlapping cases between FCAS and MWS, or MWS and CINCA exist as well. More than 50 CAPS-associated missense mutations have been reported and, of note, most of them are clustered in the NOD domain of NLRP3 [11]. As for the mechanism by which those mutations activate NLRP3 leading to autoinflammation, Lee *et al*. have recently noticed the role of the calcium-sensing receptor that regulates NLRP3 inflammasome through Ca^2+^ and cAMP [12], but other mechanisms might be contributed as well.

Caspase recruitment domain-containing protein 8 (CARD8), also called tumor-up-regulated CARD-containing antagonist of caspase nine (TUCAN) or CARD inhibitor of NF-κB-activating ligands (Cardinal), is a member of the CARD family, and is composed of an N-terminal function to find (FIIND) domain and a C-terminal CARD domain. In a pioneering study by Razmara *et al*. [13], CARD8 was shown to interact physically with caspase-1 through the CARD-CARD homophilic interaction and to negatively regulate activation of caspase-1. On the other hand, Agostini *et al*. [3] showed an interaction between the FIIND domain of CARD8 and the NOD domain of NLRP3, and they proposed a model in which NLRP3 inflammasome consists of a complex of NLRP3, ASC, caspase-1 and CARD8. Subsequently, some review articles adopted the model of an NLRP3 inflammasome including CARD8 [14-16] while others excluded CARD8 [17-19]. Thus, the association of CARD8 protein with NLRP3 and its function in the inflammasome remain obscure.

Here, we show that CARD8 plays a role as a negative regulator of NLRP3 inflammasome through its binding with NLRP3. CAPS-associated mutant NLRP3 was, however, unable to bind to CARD8.

## Methods

### cDNA cloning of inflammasome components

The study protocols using human blood cells were approved by the Medical Research Ethics Committee for Genetic Research, Tokyo Medical and Dental University. Blood samples were obtained from healthy volunteers after obtaining written informed consent, and we did not use samples from patients in this study. RNA was isolated from peripheral blood leukocytes by the acid guanidinium thiocyanate-phenol-chloroform method [20]. cDNA was synthesized from total RNA with SuperScript III reverse transcriptase (Invitrogen, Carlsbad, CA, USA) using random hexamer as a primer. The entire coding sequence of NLRP3, ASC, procaspse-1, proIL-1β and CARD8 (the T48 isoform, amino acids 1 to 431), truncated coding sequences of CARD8 (the FIIND domain, amino acids 1 to 346, or the CARD domain, amino acids 321 to 431) were amplified by PCR using modified PCR primers based on the cDNA sequences from GeneBank (NLRP3, AF410477; ASC, AB023416; procaspse-1, M87507; proIL-1β, BC008678; and CARD8, AF322184). The sequences were confirmed with an ABI Prism 3100 Genetic Analyzer (Applied Biosystems, Foster City, CA, USA). The C-terminus Flag (−DYKDDDDK), GFP, and DsRed (Clontech, Palo Alto, CA, USA) tags were used to generate fusion proteins. cDNAs of proIL-1β, ASC and fluorescent fusion proteins were subcloned into the pTargeT vector (Promega, Mannheim, Germany). NLRP3, NLRP3-Flag, CARD8, CARD8-Flag, FIIND-Flag, CARD-Flag and procasapse-1 cDNAs were subcloned into the pcDNA3.1 vector (Invitrogen).

### Polymorphism of CARD8

DNA samples were collected from healthy Japanese donors using Puregene (Gentra, Big Lake, MN, USA), and the CARD8 was amplified by PCR using the following primers: sense: 5′-GATGGAGTCGTAGGGGCCTGAG-3′ and antisense: 5′-CTCCCTCATCAGGGGCTTCACG-3′. c.30 T > A (rs2043211) variant was detected by sequencing.

### Immunoprecipitation of transient transfectants

HEK293 cells were provided by the RIKEN BRC (Tsukuba, Japan) through the National Bio-Resource Project of the Ministry of Education, Culture, Sports, Science, and Technology (MEXT) Japan. The cells were cultured in DMEM supplemented with 10% heat-inactivated fetal bovine serum, and transfected with expression plasmids pcDNA or pTargeT using Lipofectamine LTX (Invitrogen) according to the manufacturer’s protocol. At 24 hours after transfection, cells were lysed with ice-cold lysis buffer (50 mM Tris-HCl, 150 mM KCl, 1% NP-40, 0.5% sodium deoxycholate, 0.1% SDS, and 1 mM phenylmethylsulfonyl fluoride (PMSF), pH 8.0) and solubilized by a sonicator. The cell lysates were clarified by centrifugation for 10 minutes at 16,000 g and incubated for 60 minutes at 4°C with Anti-Flag M2 affinity gel (Sigma, St Louis, MO, USA). The gels were washed four times with lysis buffer and the final precipitates were subjected to Western blotting analysis using the following antibodies: anti-FLAG (Sigma), anti-CARD8 (Santa Cruz Biotechnology, Santa Cruz, CA, USA), anti-NLRP3 (nalpy3-b, Abcam, Cambridge, UK), anti-ASC (MBL, Nagoya, Japan), anti-caspase-1 (Santa Cruz Biotechnology), anti-GAPDH (Sigma), anti-IL-1β (Santa Cruz Biotechnology), and anti-cleaved IL-1β (Cell Signaling Technology, Beverly, MA, USA).

### Determination of IL-1β secretion from HEK293 cells

Cells were plated on 12-well plates and transfected with cDNAs. At 24 hours after transfection, supernatants were replaced with fresh medium and cells were incubated for another 24 hours. Then, the second supernatants and pellets were subjected to ELISA and Western blotting, respectively. ELISA was performed using Human IL-1β/IL-1 F2 Immunoassay ELISA Kit (R&D Systems, Minneapolis, MN, USA). In some experiments, secreted IL-1β during the two-hour incubation in serum-free medium (Opti-MEM, Gibco, Carlsbad, CA, USA), with which supernatants were replaced at 24 hours after transfection, was detected by Western blotting as well.

### Quantification of specks

HEK293 cells were plated on multi-well glass bottom dishes (Matsunami, Kishiwada, Japan) and transfected with the plasmids by lipofection. Twenty-four hours later, fluorescent images were obtained using a confocal laser scanning microscope FV500 (Olympus, Tokyo, Japan), and the percentage of speck-positive cells was calculated as the number of speck-positive cells divided by the total number of transfected cells.

### Immunoprecipitation of endogenous proteins

Peripheral blood mononuclear cells (PBMCs, 1 × 10^7^ cells/6 cm dish) from healthy volunteers were cultured in RPMI-1640 medium supplemented with 10% heat-inactivated fetal bovine serum, and primed with 25 ng/ml lipopolysaccharide (LPS, *Escherichia coli* 0111:B4, Sigma) for three hours. After the supernatant was replaced by fresh medium or Opti-MEM, cells were stimulated with 1.5 mM adenosine triphosphate (ATP) for one hour, then the supernatants were assayed for IL-1β ELISA or Western blotting, respectively. In addition, whole cell extracts were prepared with ice-cold W buffer (20 mM HEPES-KOH, 10 mM KCl, 1.5 mM MgCl_2_, 1 mM Na-EDTA, 1 mM Na-EGTA, 0.1 mM PMSF and protease inhibitor cocktail (Sigma), pH 7.5) and solubilized by a sonicator [8]. The cell lysates were clarified by centrifugation for 10 minutes at 16,000 g and incubated overnight at 4°C with anti-NLRP3 antibody. The reaction mixtures were then incubated for 60 minutes at 4°C with protein G-Sepharose to precipitate the antigen-antibody complexes. The precipitates were washed five times with W buffer and subjected to Western blotting analysis.

### RNAi knockdown of CARD8

Human monocytes were isolated from peripheral blood cells using RosetteSep (Stemcell Technologies, Vancouver, BC, Canada). Human monocyte-derived macrophages (HMDMs) were differentiated after incubation of the monocytes for seven days in RPMI-1640 medium supplemented with 10% human serum, 10% heat-inactivated fetal bovine serum and 50 ng/ml GM-CSF (PeproTech, Rocky Hill, NJ, USA). The human CARD8-specific siRNA (siCARD8-1; 5′-CCUCUUAUGCUUCUAAAGUCU-3′ and 5′-ACUUUAGAAGCAUAAGAGGAA-3′) was synthesized by Sigma. Another set of CARD8-specific siRNA (siCARD8-2; 5′-GAGCCUUUCUAUGCUGUCCUGGAAA-3′ and 5′-UUUCCAGGACAGCAUAGAAAGGCUC-3′) and negative control duplex were purchased from Invitrogen. HMDMs were transiently transfected with each siRNA (50 nM) using Lipofectamine RNAiMax (Invitrogen). At four days after transfection, knockdown efficiency was evaluated by real-time quantitative RT-PCR. Then the cells were primed with 10 ng/ml LPS for three hours followed by stimulation with 1.5 mM ATP for one hour, and IL-1β in the culture supernatants was measured by ELISA. In parallel, ATP-stimulated cells were labeled with a FAM-YVAD-fmk FLICA caspase-1 assay kit (Immunochemistry, Bloomington, IN, USA), and analyzed by flow cytometry to evaluate the activity of caspase-1.

### Statistical analysis

Student’s unpaired *t-*tests were performed for statistical comparisons, and a *P-*value of less than 0.05 was considered significant.

## Results

### Interaction of CARD8 with NLRP3

**Figure 1:**
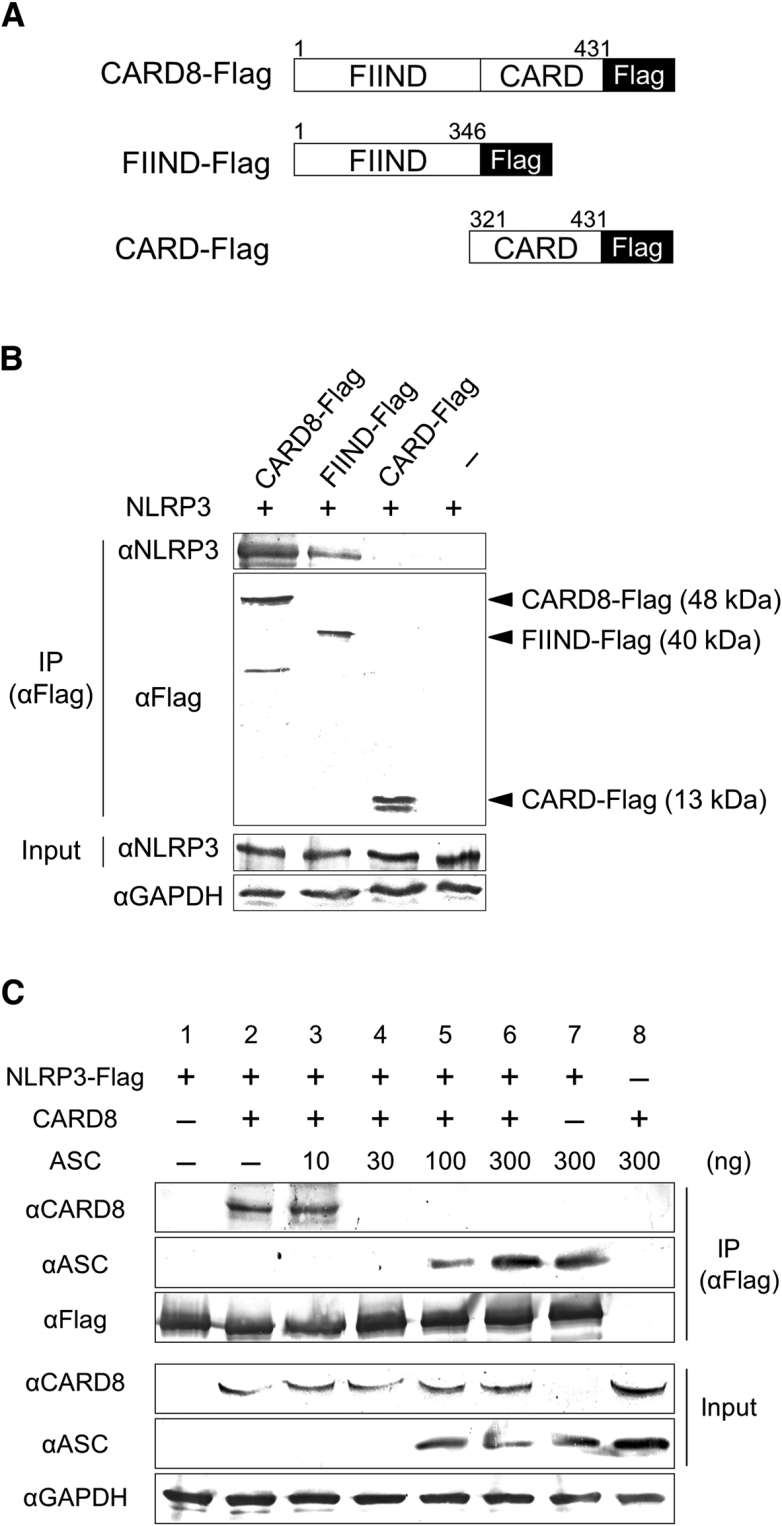
**Interaction of CARD8 with full length NLRP3. (A)** Diagrammatic representation of the dual domain structure of caspase recruitment domain-containing protein 8 (CARD8). **(B)** Human Embryonic Kidney (HEK)293 cells were cotransfected with 500 ng each of expression plasmid for NLRP3 and either CARD8-Flag, function-to-find (FIIND)-Flag, CARD-Flag or an empty vector. At 24 hours after transfection, whole cell lysates were immunoprecipitated (IP) with anti-Flag antibody and analyzed by Western blotting. The total amount of the plasmid DNA in all transfection reactions was kept constant by adding the empty vector plasmid. **(C)** HEK293 cells were transfected with expression plasmids for NLRP3-Flag (500 ng), CARD8 (500 ng) and apoptosis-associated speck-like protein containing a CARD (ASC) (10, 30, 100 or 300 ng), and analyzed as above.

To study the interactions between CARD8 and NLRP3, expression plasmid harboring Flag-tagged full length CARD8, the FIIND domain or the CARD domain of CARD8 was constructed (Figure 1A). Each expression plasmid was introduced into HEK293 cells with expression plasmid of NLRP3 and immunoprecipitated using anti-Flag antibody. As shown in Figure 1B, full length CARD8 and the FIIND domain, but not the CARD domain were immunoprecipitated with NLRP3, suggesting that CARD8 interacts with NLRP3 through the FIIND domain. To rule out the possibility that the tagging might affect the results, we carried out similar experiments using Flag-tagged NLRP3 and CARD8, instead of NLRP3 and Flag-tagged CARD8, and obtained similar results (Figure 1C, lane 2).

Next, to study the effect of ASC on the interaction of NLRP3 with CARD8, ASC expression plasmid was introduced to the cells. The interaction between CARD8 and NLRP3 was inhibited by the coexpression of ASC in a dose-dependent manner (lanes 3 to 6). When 100 ng or higher doses of ASC-expression plasmid were transfected, NLRP3 was coprecipitated with ASC rather than with CARD8 (lanes 5 to 7). Thus, the interaction of ASC with NLRP3 seemed to be stronger than that of CARD8 with NLRP3 in this condition.

### Suppression of NLRP3 inflammasome by expression of CARD8

**Figure 2:**
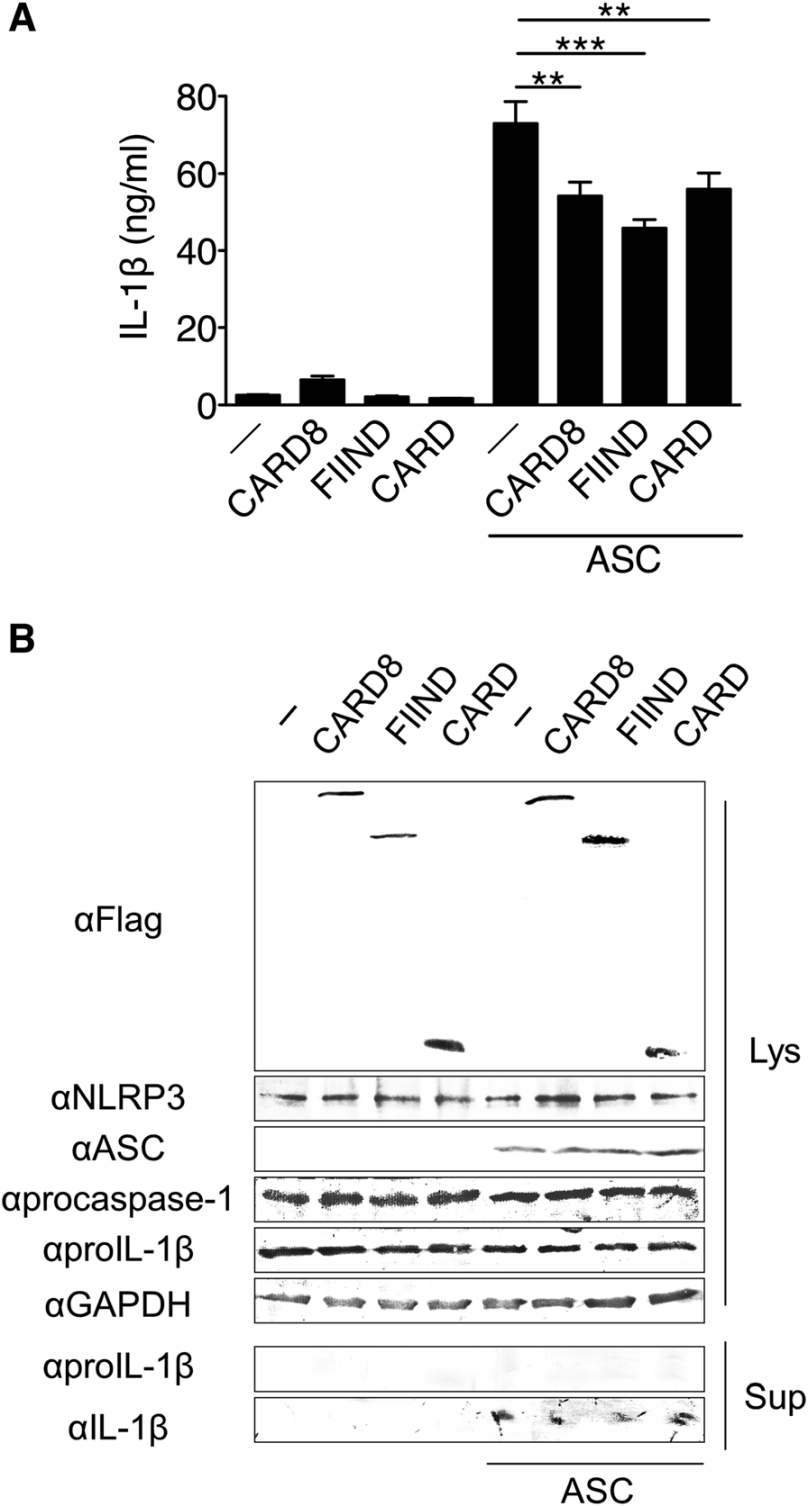
**Suppression of NLRP3 inflammasome by expression of CARD8. (A)** Human Embryonic Kidney (HEK)293 cells were transfected with expression plasmids for proIL-1β (200 ng), NLRP3 (50 ng), procaspse-1 (10 ng), apoptosis-associated speck-like protein containing a CARD (ASC) (25 ng) and either caspase recruitment domain-containing protein 8 (CARD8)-Flag, function-to-find (FIIND)-Flag, CARD-Flag or empty vector (100 ng each). At 24 hours after transfection, the supernatants were replaced by fresh medium and incubated for another 24 hours; thereafter, the second supernatants were subjected to ELISA. Each column represents the mean ± SD (n = 4), **P <0.005, and ***P <0.001 vs. no CARD8 component. **(B)** HEK293 cells were transfected with the same set of plasmids as A. A total of 24 hours after transfection, or after a further two-hour incubation in serum-free medium, cell lysates (Lys) or supernatants (Sup), respectively, were analyzed by Western blotting.

When HEK293 cells were transfected with every inflammasome component, that is, NLRP3, ASC, procaspase-1 and proIL-1β, large amounts of IL-1β were secreted spontaneously. To examine the effect of CARD8 on activation of the NLRP3 inflammasome, the expression construct of CARD8 was cotransfected, which resulted in significant reduction of IL-1β secretion (Figure 2A). The FIIND domain and the CARD domain also inhibited IL-1β secretion suggesting that not only the CARD8-procaspase-1-interaction as shown by other investigators [13], but also the CARD8-NLRP3-interaction contributes to the reduction of IL-1β secretion. In this experiment, production of IL-1β and expression of inflammasome components were confirmed by Western blot (Figure 2B).

### Suppression of NLRP3-speck formation by CARD8

**Figure 3:**
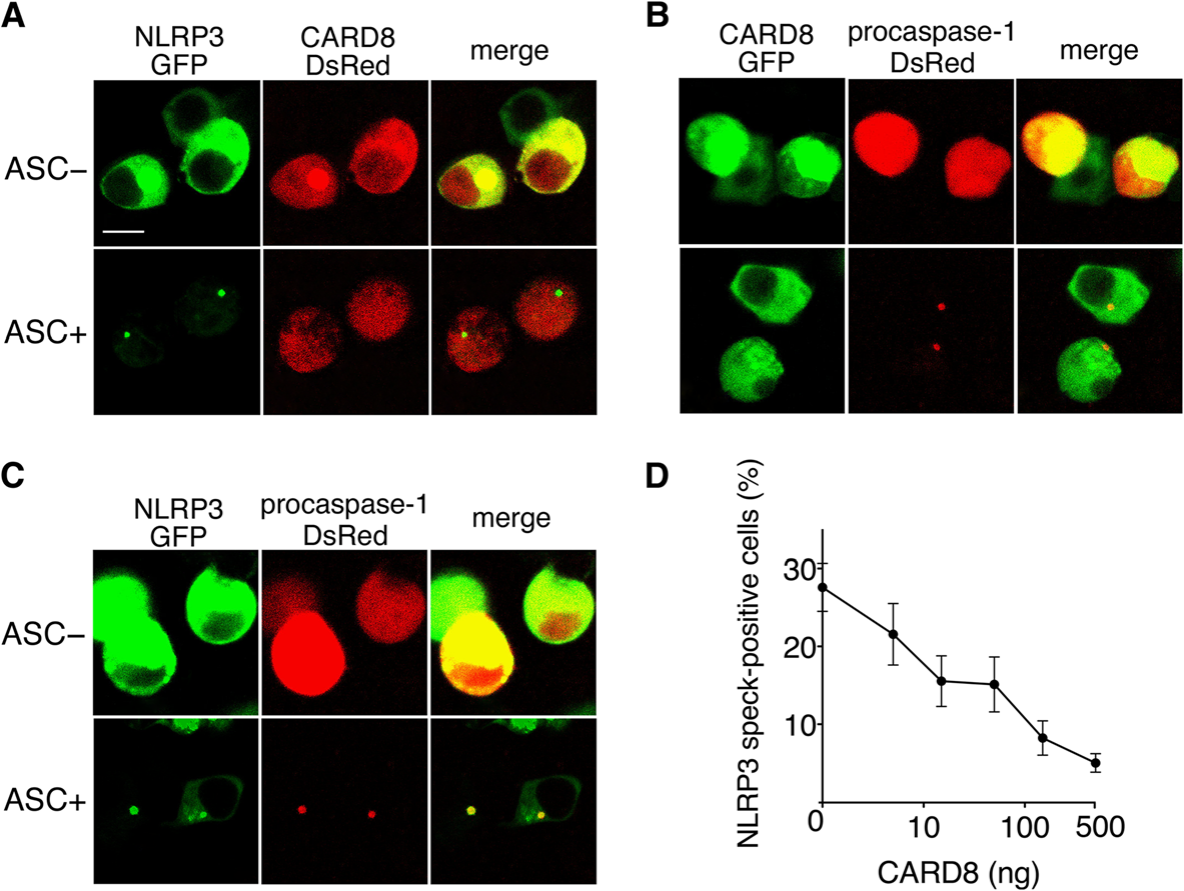
**Suppression of NLRP3-speck formation by CARD8. (A)** Human Embryonic Kidney (HEK)293 cells were cotransfected with expression plasmid constructs of NLRP3-GFP, caspase recruitment domain-containing protein 8 (CARD8)-DsRed, procaspase-1, and either apoptosis-associated speck-like protein containing a CARD (ASC) or an empty vector. After 24 hours, green and red fluorescent signals were detected by a confocal laser scanning microscope. The scale bar represents 10 μm. **(B)** The same as A except for using NLRP3, CARD8-GFP and procaspase-1-DsRed. **(C)** The same as A, except for using NLRP3-GFP, CARD8 and procaspase-1-DsRed. **(D)** HEK293 cells were cotransfected with expression plasmids for NLRP3-GFP (30 ng), ASC (5 ng), procaspase-1 (2.5 ng), proIL-1β (30 ng) and CARD8 (5, 15, 50, 150 or 500 ng) or an empty vector. At 24 hours after transfection, the percentage of NLRP3-GFP-speck positive cells in the transfected cells was determined. Data represent the mean ± SD (n = 9).

ASC is known to recruit NLRP3 and caspase-1 to form a speck-like assembly of the proteins [6,21,22]. To examine the effect of CARD8 on speck formation in the presence of ASC, NLRP3 and procaspase-1, fluorescent fusion proteins were expressed in HEK293 cells. After transfection, we observed GFP and DsRed in more than half of the cells. In representative microscopic fields, NLRP3-GFP was localized in the cytoplasm, while CARD8-DsRed was localized in both the nucleus and cytoplasm (Figure 3A, upper panel). When ASC was co-expressed in the cell, NLRP3-GFP formed speck-like aggregates, while CARD8-DsRed did not (Figure 3A, lower panel). CARD8-GFP and procaspase-1-DsRed were localized in both the nucleus and cytoplasm in the absence of ASC (Figure 3B, upper panel). Coexpression of ASC induced speck-like aggregates containing procaspase-1-DsRed, but not CARD8-GFP, in the cytoplasm (Figure 3B, lower panel). ASC-included speck-like aggregates were confirmed to contain NLRP3-GFP and procaspase-1-DsRed (Figure 3C, lower panel). These results suggest that overexpression of ASC recruits both NLRP3 and procaspase-1 to form the inflammasome, which does not contain CARD8.

Next, to investigate the role of CARD8, the number of speck-positive cells in the expression of different dosages of CARD8 was counted. When cells were transfected with NLRP3-GFP, ASC, procaspase-1 and proIL-1β, the percentage of NLRP3-speck positive cells was 27.1 ± 2.9% (mean ± SD). However, the number of speck positive cells was reduced by coexpression of CARD8 in a dose-dependent manner (Figure 3D). Transfection of ASC alone has been known to form speck [23,24], but coexpression of CARD8 had no effect on the number of ASC-speck positive cells (data not shown). These results suggest that CARD8 negatively regulates formation of NLRP3 inflammasome, although the final product of the inflammasome does not contain CARD8. Therefore, we speculate that expression of CARD8 leads to a reduced amount of inflammasome formation, and this results in a reduced amount of IL-1β secretion by the cells than those without CARD8 expression.

### No interaction between CARD8 and CAPS-associated NLRP3 mutants

**Figure 4:**
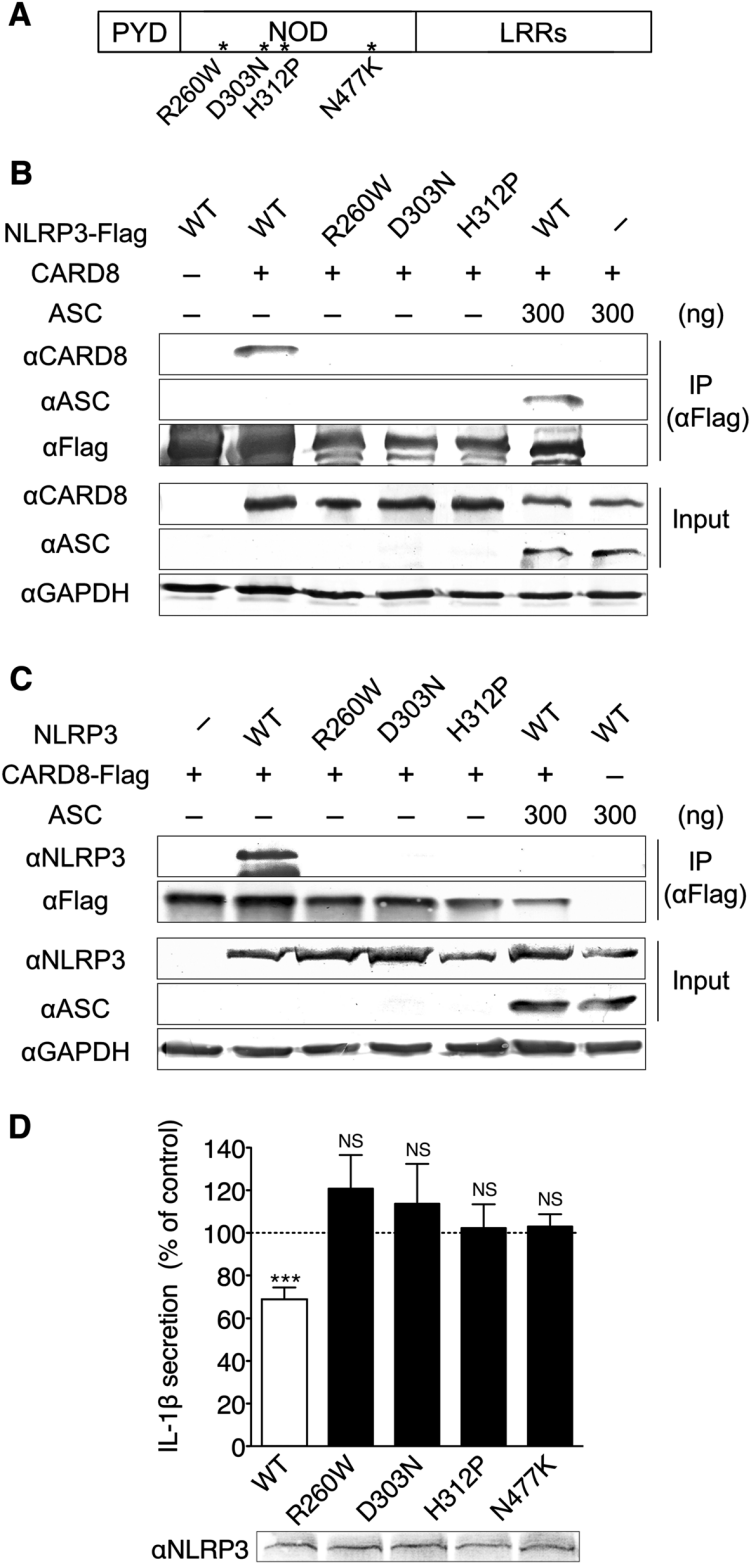
**CARD8 fails to bind CAPS-associated NLRP3 mutants. (A)** Diagrammatic representation of the three-domain structure of NLRP3 with asterisks indicating the location of mutations. **(B)** Human Embryonic Kidney (HEK)293 cells were transfected with expression plasmids (500 ng each) for NLRP3-Flag (wild type (WT), R260W, D303N or H312P) and caspase recruitment domain-containing protein 8 (CARD8) with or without apoptosis-associated speck-like protein containing a CARD (ASC) (300 ng). At 24 hours after transfection, expression of each protein (Input) was confirmed by Western blotting. Simultaneously, the whole cell lysates were the immunoprecipitated (IP) with anti-Flag antibody and analyzed by Western blotting. **(C)** Similar experiments as above were carried out, but CARD8 was Flag-tagged in place of NLRP3. **(D)** HEK293 cells were transfected with expression plasmids of proIL-1β (200 ng), NLRP3 (WT, R260W, D303N, H312P or N477K) (50 ng), procaspase-1 (10 ng), ASC (25 ng) and CARD8 or an empty vector (100 ng). At 24 hours after transfection, the supernatants were replaced by fresh medium and incubated for another 24 hours; thereafter, the second supernatants were subjected to ELISA and pellets were analyzed by Western blotting. Levels of IL-1β secretion from transfectants were expressed as a relative percentage compared with those from cells with each NLRP3 without CARD8. Each column represents the mean ± SD (n = 3), ***P <0.001 vs. no CARD8. NS, not significant vs. no CARD8.

To test the interaction of CARD8 with CAPS-associated NLRP3 mutants, we engineered NLRP3 expression plasmids containing the mutations found in CAPS patients. The missense mutation of R260W was reported in FCAS and MWS, D303N in MWS and CINCA, and N477K in CINCA [25]. H312P is a rare mutation which was first found in a patient with MWS in our hospital [26] (Figure 4A). In the absence of ASC, wild type NLRP3-Flag was precipitated with CARD8 (Figure 4B, lane 2). In contrast, Flag-tagged NLRP3 with R260W, D303N or H312P mutation failed to bind to CARD8 (lanes 3 to 5). Similar results were observed when we carried out the experiments using CARD8-Flag and NLRP3, instead of CARD8 and NLRP3-Flag (Figure 4C). To study whether the effect of CARD8 on the inflammasome is different between the inflammasome containing wild type NLRP3 and mutant NLRP3, we compared the IL-1β concentration in the culture medium (Figure 4D). IL-1β secretion from the cells transfected with wild type NLRP3 was significantly inhibited by the coexpression of CARD8, which was consistent with the results shown in Figure 2. In contrast, CARD8 did not inhibit IL-1β secretion from the cells transfected with mutant NLRP3 in any of the cases we tested.

### Interaction of endogenous CARD8 and NLRP3

**Figure 5:**
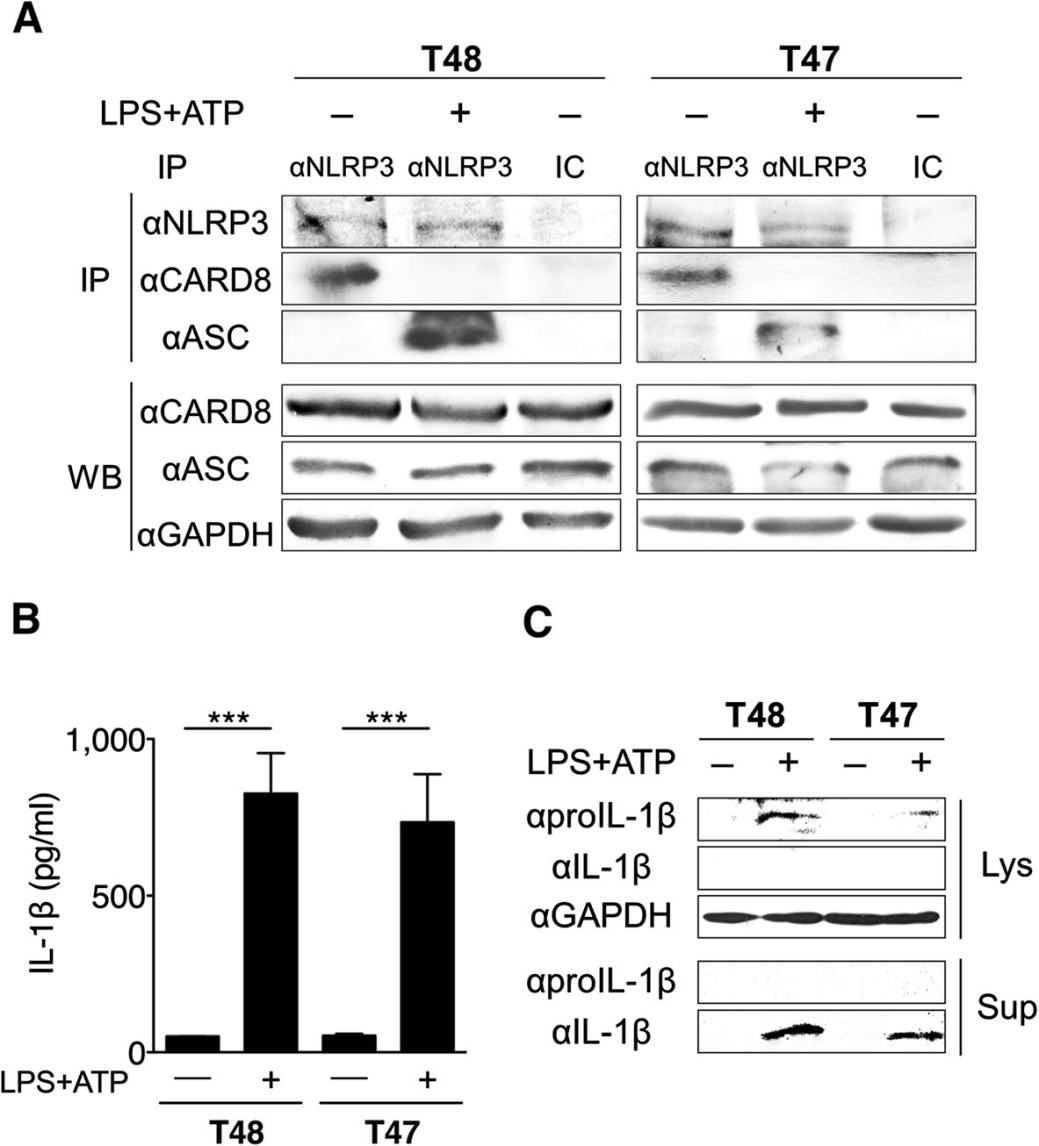
**Endogenous CARD8 interacts with NLRP3 irrespective of the isoform. (A)** Cell lysates of peripheral blood mononuclear cells (PBMCs) from healthy volunteers with the T48 isoform or T47 isoform before and after stimulation by lipopolysaccharide (LPS) and adenosine triphosphate (ATP) were analyzed by immunoprecipitation (IP) and Western blotting. IC: isotype-matched control antibody. **(B)** IL-1β in supernatants from cells stimulated as in A was measured by ELISA. Each column represents the mean ± SD (n = 4), ***P <0.001. **(C)** IL-1β in lysates (Lys) and supernatants (Sup) from the cells stimulated as in A was analyzed by Western blotting. CARD8, caspase recruitment domain-containing protein 8.

Apart from the transfected cells, we were interested in examining the interaction between endogenous CARD8 and NLRP3. The CARD8 gene is known to have a single nucleotide polymorphism (rs2043211) that results in an A > T transversion. Genotype A/A expresses the T48 isoform, and genotype T/T expresses the T47 isoform in which the first 25 amino acids at the N-terminus of T48 are replaced by an alternative 20 amino acids [27]. The majority (approximately 90%) of the population in Germany, Hungary and the Netherlands are reported to have the T48 isoform [28]. In monocytes and macrophages, it is known that LPS priming induces proIL-1β synthesis, and the following ATP stimulation activates NLRP3 via the P2X7 receptor [29,30]. The interaction between endogenous CARD8 and NLRP3 was tested using the PBMCs of healthy volunteers. In the resting state, NLRP3 was precipitated with CARD8, but not with ASC (Figure 5A). When cells were primed with LPS followed by ATP stimulation, however, NLRP3 was precipitated with ASC instead of CARD8 (Figure 5A), and high amounts of IL-1β were secreted into the culture medium (Figure 5B, C). Similar results were observed in the T48 and T47 isoforms.

### Enhanced ATP-induced IL-1β secretion by CARD8-knockdown

**Figure 6:**
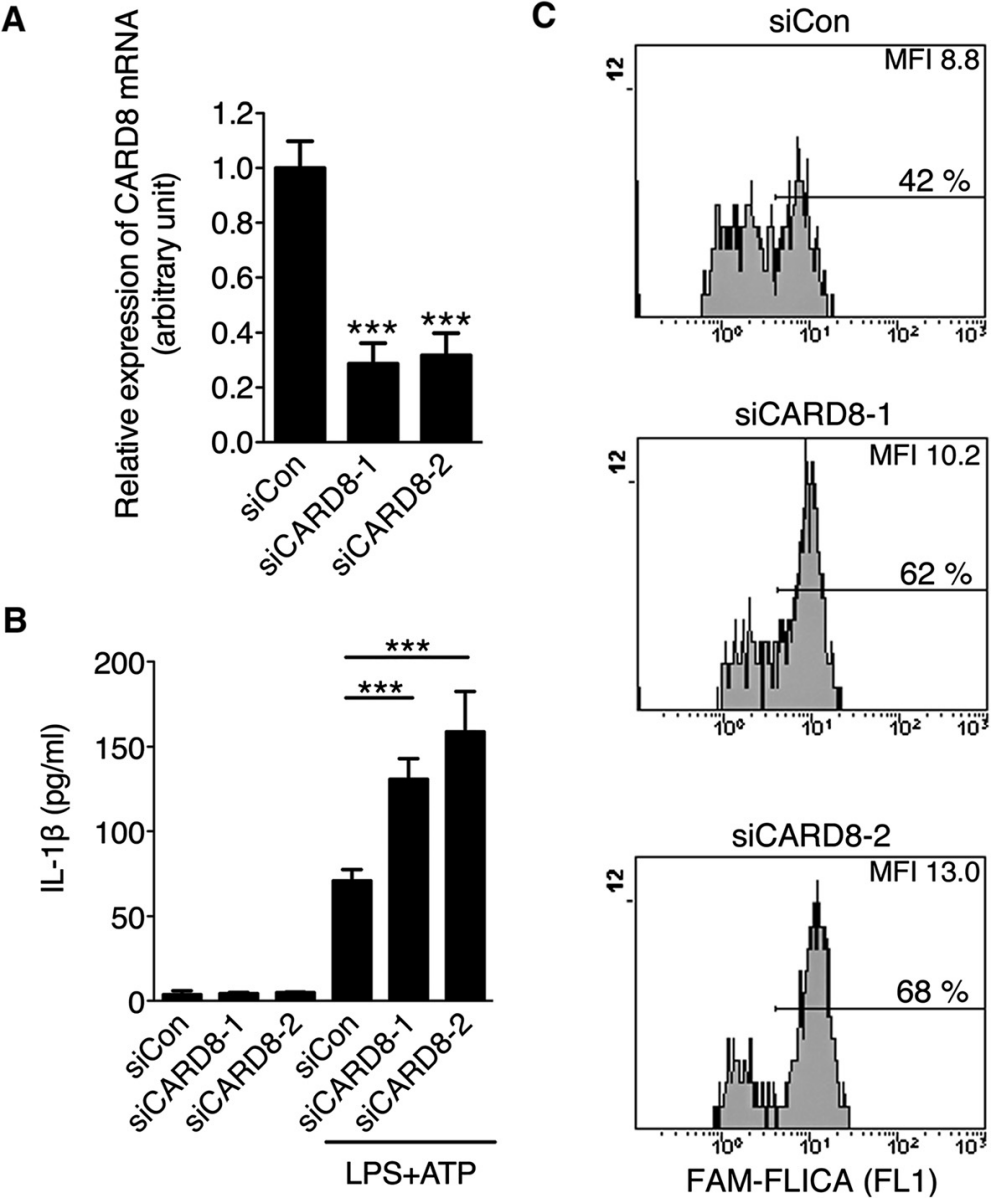
**CARD8-knockdown facilitates ATP-induced IL-1β secretion. (A)** Caspase recruitment domain-containing protein 8 (CARD8) targeting (siCARD8-1, siCARD8-2) or control (siCon) RNA (50 nM) was transfected in human monocyte-derived macrophages (HMDMs). After stimulation of the cells with lipopolysaccharide (LPS), efficiency of the knockdown was evaluated by qPCR. **(B)** CARD8-knockdown HMDMs were stimulated with LPS and adenosine triphosphate (ATP), and secreted IL-1β was measured by ELISA. **(C)** CARD8-knockdown HMDMs were stimulated with LPS and ATP, and activation of caspase-1 was determined by flow cytometry using a fluorescence-labeled irreversible caspase-1 substrate (FLICA). The data shown are representative of three independent experiments with similar results. Each column represents the mean ± SD (n = 4), ***P <0.001.

To define the suppressive effect of endogenous CARD8 on NLRP3 inflammasome, CARD8 was knocked down in HMDMs and ATP-induced IL-1β secretion was measured. By transfection of CARD8-specific siRNA (siCARD8-1 or siCARD8-2), expression of CARD8 mRNA was significantly suppressed (Figure 6A). When these HMDMs were stimulated with LPS and ATP, CARD8-knockdown HMDMs secreted higher amounts of IL-1β than those transfected with control RNA (Figure 6B). Accordingly, in the flow cytometric analysis of the caspase-1 activity, the number of activated-caspase-1 positive cells as well as their mean fluorescence intensity was higher in the CARD8-knockdown HMDMs than those transfected with the control RNA (Figure 6C).

## Discussion

In the present study, we obtained four major findings: 1) in the HEK293 cell expression system, CARD8 interacted with wild-type NLRP3 but not with CAPS-associated mutant NLRP3; 2) CARD8 suppressed IL-1β secretion from the cells transfected with wild-type NLRP3, but not from the cells with CAPS-associated mutant NLRP3; 3) in PBMCs from healthy subjects, endogenous NLRP3 interacted with CARD8 in a resting state, but the partner changed to ASC after stimulation by LPS and ATP; and finally, 4) in HMDMs from healthy subjects, ATP-stimulated IL-1β secretion was increased by knockdown of CARD8.

Using HEK293T cells and calcium phosphate transfection methods, it has been reported that VSV-tagged CARD8 interacts with Flag-tagged truncated NLRP3 (ΔLRRs), but no interaction was detected between VSV-tagged CARD8 and Flag-tagged full-length NLRP3 [3]. In our experiments, however, CARD8 interacted with full length NLRP3. Differences in tagging, cells, culture medium and transfection methods may produce different results. We were cautious, therefore, and experiments using Flag-tagged NLRP3 were repeated using Flag-tagged CARD8, with similar results. Furthermore, we confirmed the interaction between endogenous CARD8 and NLRP3 in normal PBMCs. In the transfection study with HEK293 cells, NLRP3 did interact with CARD8, but ASC prevented the interaction in a dose-dependent manner. Moreover, HEK293 cells transfected with every component of NLRP3 inflammasome spontaneously showed speck formation and produced IL-1β. These results suggest that gene transfer itself may induce conversion of NLRP3 to activated form, to which CARD8 does not bind. Consistent with this hypothesis, endogenous NLRP3 interacted with CARD8 in PBMCs only in a resting state; after stimulation with LPS and ATP, NLRP3 interacted not with CARD8 but with ASC. CARD8 may hold NLRP3 in an inactive form until the cells encounter stimuli over a threshold level.

Families of small proteins that are called CARD-only proteins (COPs) and PYD-only proteins (POPs) have recently been known to negatively regulate inflammasome and suppress spontaneous or unnecessary activation by playing a role as decoy partners [31-33]. Similar to COPs, the CARD domain of CARD8 is reported to interact with the CARD domain of procaspase-1; these CARD domains are highly homologous to each other, resulting in suppression of autocleavage of procaspase-1 [13]. In the present study, IL-1β secretion was reduced in HEK293 cells transfected with every inflammasome component, by coexpression of the FIIND domain of CARD8, suggesting that this domain is also responsible for negative regulation of NLRP3 inflammasome by inhibiting NLRP3 oligomerization. Furthermore, CARD8-knockdown HMDMs secreted a significantly higher amount of IL-1β along with caspase-1 activation, suggesting that CARD8 plays a role as a negative regulator on the endogenous NLRP3 inflammasome as well. How much the suppressive effect of CARD8 on IL-1β secretion resulted from the interaction with NLRP3 independent of that with procaspase-1 remains to be elucidated. Analogous to this hypothesis, CARD8 has been recently reported to interact with the NOD domain of NOD2, which is one of the NLR family members, and inhibits nodosome assembly [34].

We also examined interaction of CARD8 and CAPS-associated NLRP3 mutants, but no significant interaction was detected with any mutants tested. This may result from a change of the local structure by replacement of an amino acid in the NOD domain that is normally the binding site for CARD8. As a result, CARD8 did not reduce IL-1β secretion by inflammasome containing mutant NLRP3. These results suggest that CAPS-associated mutant NLRP3 escapes the CARD8 restriction, which might be responsible for unnecessary activation of NLRP3 inflammasome in the patients.

## Conclusion

Our data support the model of NLRP3 inflammasome excluding CARD8. Until specific stimuli activate NLRP3, CARD8 holds NLRP3, and is supposed to prevent activation by subtle stimuli. Because CAPS-associated mutant NLRP3 is not protected by CARD8, it may form inflammasome without obvious stimulation.

## Abbreviations

ASC: Apoptosis-associated speck-like protein containing a CARD; ATP: Adenosine triphosphate; CAPS: Cryopyrin-associated periodic syndromes; CARD: Caspase recruitment domain; CARD8: Caspase recruitment domain-containing protein 8; Cardinal: CARD inhibitor of NF-κB-activating ligands; CIAS1: Cold-induced autoinflammatory syndrome 1; CINCA: Chronic infantile neurologic cutaneous articular syndrome; COPs: CARD-only proteins; DAMPs: Damage-associated molecular patterns; FCAS: Familial cold autoinflammatory syndrome; FIIND: Function to find; HMDMs: Human monocyte-derived macrophages; IL: Interleukin; IP: Immunoprecipitation; LPS: Lipopolysaccharide; LRRs: Leucine-rich repeats; MFI: Mean fluorescence intensity; MWS: Muckle-Wells syndrome; NLR: NOD-like receptor; NOD: Nucleotide binding oligomerization domain; PAMPs: Pathogen-associated molecular patterns; PBMCs: Peripheral blood mononuclear cells; PCR: Polymerase chain reaction; POPs: PYD-only proteins; PYD: Pyrin domain; TUCAN: Tumor-upregulated CARD-containing antagonist of caspase nine; WT: Wild-type.

## Competing interests

The authors declare that they have no competing interests.

## Authors’ contributions

All authors were involved in drafting the article or revising it critically for important intellectual content, and all approved the final version to be published. SI, YH and TK participated in study conception and design, analysis and interpretation of data. SI and YH contributed to acquisition of data.
